# End-to-End Models to Imitate Traditional Chinese Medicine Syndrome Differentiation in Lung Cancer Diagnosis: Model Development and Validation

**DOI:** 10.2196/17821

**Published:** 2020-06-16

**Authors:** Ziqing Liu, Haiyang He, Shixing Yan, Yong Wang, Tao Yang, Guo-Zheng Li

**Affiliations:** 1 Second School of Clinic Medicine Guangzhou University of Chinese Medicine Guangzhou China; 2 School of Artifical Intelligence and Information Techology Nanjing University of Chinese Medicine Nanjing China; 3 Shanghai Bright AI Co, Ltd Shanghai China; 4 Shanghai Literature Institute of Traditional Chinese Medicine Shanghai China

**Keywords:** traditional Chinese medicine, syndrome differentiation, lung cancer, medical record, deep learning, model fusion

## Abstract

**Background:**

Traditional Chinese medicine (TCM) has been shown to be an efficient mode to manage advanced lung cancer, and accurate syndrome differentiation is crucial to treatment. Documented evidence of TCM treatment cases and the progress of artificial intelligence technology are enabling the development of intelligent TCM syndrome differentiation models. This is expected to expand the benefits of TCM to lung cancer patients.

**Objective:**

The objective of this work was to establish end-to-end TCM diagnostic models to imitate lung cancer syndrome differentiation. The proposed models used unstructured medical records as inputs to capitalize on data collected for practical TCM treatment cases by lung cancer experts. The resulting models were expected to be more efficient than approaches that leverage structured TCM datasets.

**Methods:**

We approached lung cancer TCM syndrome differentiation as a multilabel text classification problem. First, entity representation was conducted with Bidirectional Encoder Representations from Transformers and conditional random fields models. Then, five deep learning–based text classification models were applied to the construction of a medical record multilabel classifier, during which two data augmentation strategies were adopted to address overfitting issues. Finally, a fusion model approach was used to elevate the performance of the models.

**Results:**

The F1 score of the recurrent convolutional neural network (RCNN) model with augmentation was 0.8650, a 2.41% improvement over the unaugmented model. The Hamming loss for RCNN with augmentation was 0.0987, which is 1.8% lower than that of the same model without augmentation. Among the models, the text-hierarchical attention network (Text-HAN) model achieved the highest F1 scores of 0.8676 and 0.8751. The mean average precision for the word encoding–based RCNN was 10% higher than that of the character encoding–based representation. A fusion model of the text-convolutional neural network, text-recurrent neural network, and Text-HAN models achieved an F1 score of 0.8884, which showed the best performance among the models.

**Conclusions:**

Medical records could be used more productively by constructing end-to-end models to facilitate TCM diagnosis. With the aid of entity-level representation, data augmentation, and model fusion, deep learning–based multilabel classification approaches can better imitate TCM syndrome differentiation in complex cases such as advanced lung cancer.

## Introduction

Lung cancer is a source of hardship worldwide, with high incidence and mortality [1,2]. According to cancer registration data collected by the Chinese National Central Cancer Registry, over 650,000 people were diagnosed with lung cancer in 2011 [3]. Standard treatment options for lung cancer are surgery, radiotherapy, and chemotherapy [4]. However, patients with low health status, such as patients in advanced stages, tend to have low tolerability of regular treatments [5]. As a respected component of traditional Chinese medicine (TCM), Chinese herbal medicine possesses the advantages of availability, efficacy, and lower toxicity than chemotherapy and radiotherapy [6]. Moreover, its benefits and underlying mechanisms in cancer therapy have been elucidated by a body of research [7-10]. After long-term practice, clinical evidence has also shown that TCM for cancer therapy can stabilize tumor lesions, enhance quality of life, and prolong survival [11,12]. More than 1 billion TCM treatments are performed in China every year according to the China Public Health Statistical Yearbook [13], and this figure is expected to increase further; meanwhile, the number of high-level TCM experts is insufficient to support the vast need for TCM.

The efficacy of TCM treatment is based on syndrome differentiation, a diagnosis method in TCM that stratifies patients’ conditions with their respective disease and then guides the choice of TCM intervention [14]. Master TCM syndrome differentiation is an intricate and time-consuming process. Because the aptitudes of clinicians vary, it can be difficult to maintain stable efficacy when treating a given disease. Therefore, differentiating syndromes when confronted with complex and aggressive cancers can be challenging [15].

From the perspective of informatics, the TCM syndrome differentiation procedure can be regarded as supervised classification. Statistical machine learning algorithms have been applied to establish TCM diagnosis models [16], such as naïve Bayes [17], decision tree [18], support vector machine [19], and K-nearest neighbor [20]. However, in clinical practice, patients can concurrently suffer from multiple diseases. In this case, TCM diagnoses of several syndromes can coexist. In this circumstance, multilabel classifiers are applied to address a problem in which a set of syndromes designates one sample. Utilizing inquiry diagnosis, Liu et al [21] constructed coronary heart disease syndrome differentiation models through various multilabel learning algorithms. Their experiment showed that the multilabel k-nearest neighbor algorithm outperformed other algorithms. Wang et al [22] formulated chronic fatigue syndrome differentiation as a multilabel learning task. Combining random forest, conformal prediction framework, and problem transformation methods, they established a reliable diagnostic tool with large-scale confidence levels from 80%-100%.

In accordance with the universal approximation theorem, a deep neural network with a given number of hidden layers should be able to approximate any function that exists between input and output [23]. With the proliferation of neural networks and the growing body of TCM clinical records, syndrome differentiation modeling approaches adopting deep neural networks have become a trend. Liu et al [24] collected 919 TCM inquiry diagnosis scales and established a deep belief network based on a multilabel model for chronic gastritis TCM syndrome diagnosis. This network demonstrated superior performance for all five evaluation measures. Moreover, the average precision was 2% higher than that of the second best performing algorithm. Xu et al [25] designed an artificial neural network with 10 hidden layers for chronic obstructive pulmonary disease TCM syndrome differentiation. According to the Global Initiative for Chronic Obstructive Lung Disease, 18,471 structured TCM outpatient medical records were separated into 4 subgroup datasets, and the subgroup artificial neural network models were trained. The evaluation indicated that subgroup syndrome differentiation models outperformed the full-group model.

Due to the flexibility and compactness of TCM clinical records, datasets used in syndrome classifier training tend to be constructed manually from free-text medical records to reproduce the syndrome differentiation process. This is a labor-intensive task that requires extensive medical expertise; some information loss is inevitable [26,27]. Considering the inaccessibility of TCM literature, Hu et al [28] modeled yin-yang syndrome differentiation as a text classification task. By employing a convolutional neural network (CNN) and the fastText classifier, two sets of experiments were conducted. The results showed that the CNN system using 5-gram characters as its inputs was the most accurate.

The aforementioned studies denote that weighted mathematical logic operation–based models can be used for intelligent TCM syndrome differentiation. However, symptom classification and the determination of diagnostic thresholds are subjective; thus, many adjustments are needed. Moreover, disputes persist regarding the objectification and correction of the weighted coefficient. Furthermore, most TCM syndrome differentiation models assume that input variables such as symptoms are mutually independent. This assumption does not conform to clinical observations.

To better generalize the experience of TCM experts, we modeled syndrome differentiation for lung cancer in the form of medical record text classification. As in previous research that seeks to uncover relationships between symptoms and herbs and between syndromes and prescriptions [29], this work models TCM syndrome differentiation for lung cancer and the procedure for TCM lung cancer diagnosis. The contributions of this work are as follows:

1. Syndrome factors, rather than the syndromes themselves, are adopted and standardized as labels to address the redundancy and changeability of TCM syndromes.
2. Two encoding gradients represent medical entities by applying Bidirectional Encoder Representations from Transformers (BERT) and conditional random fields (CRF) methods.
3. A data fusion approach capitalizes on all models to improve performance by building ensemble models.
4. Two data augmentation approaches were used to overcome the difficulties of ill-posed problems of samples and overfitting.

## Methods

### Study Design

**Figure 1:**
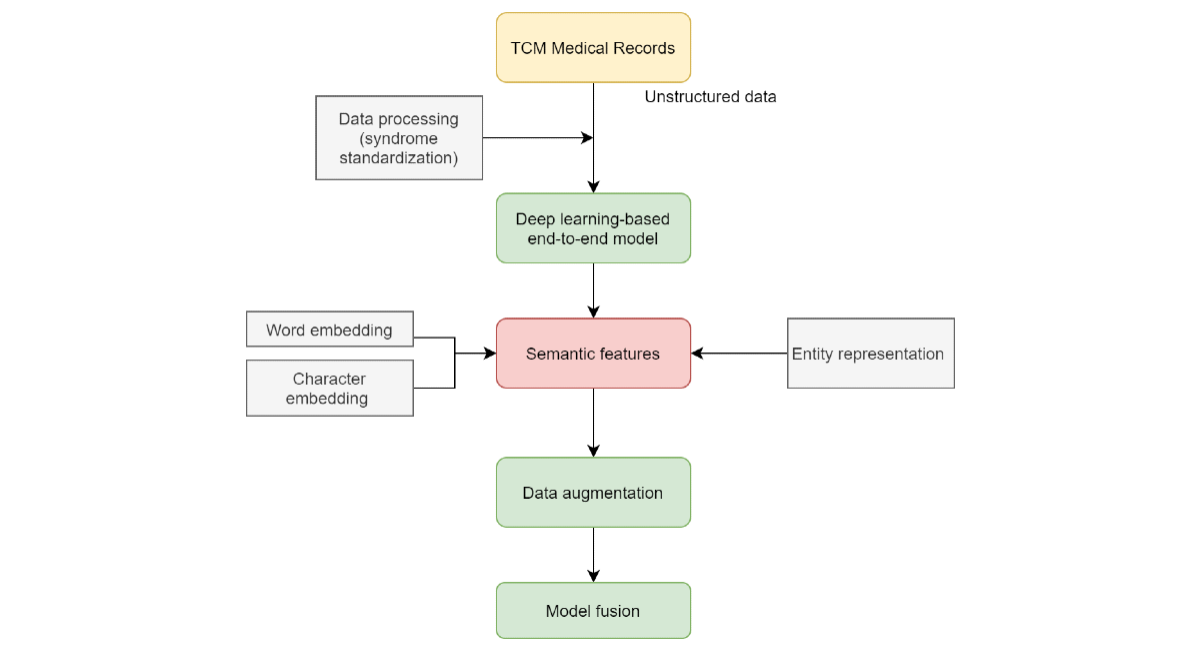
Framework of the end-to-end traditional Chinese medicine syndrome differentiation model.

Our work can be divided into entity-level representation learning and multilabel classifier modeling. As classified objects, TCM syndromes were split into sets of syndrome factors according to the principle of TCM syndrome factor differentiation [30]. Medical record texts were sent to the established networks to learn words and encode characters; then, the titles were extracted. Considering the difficulties of ill-posed problems of samples and overfitting, two data augmentation approaches were added. Finally, a model fusion framework was constructed. The optimum parameters for each deep learning algorithm and the best-performing algorithm were selected separately through the validation set. The framework is shown in Figure 1.

### Entity-Level Representation

We employed the BERT-CRF framework [31,32] to build entity-level representation. We used both character and word-row texts as input for the pre-trained BERT model to obtain semantic coding. We then saved it as a code list according to the word/character sequence. Meanwhile, a CRF architecture was assembled as the output layer to predict the text sequence labels and recognize the medical entities. Based on the semantic code list and the recognized entities, we generated entity-level representation with concatenating individual code in the order of the defined code list. We believed that the entity-level strategy would exploit the prior knowledge of TCM medical information that was implicitly learned during training. Multilabel classifier modelling was used for syndrome differentiation.

**Figure 2:**
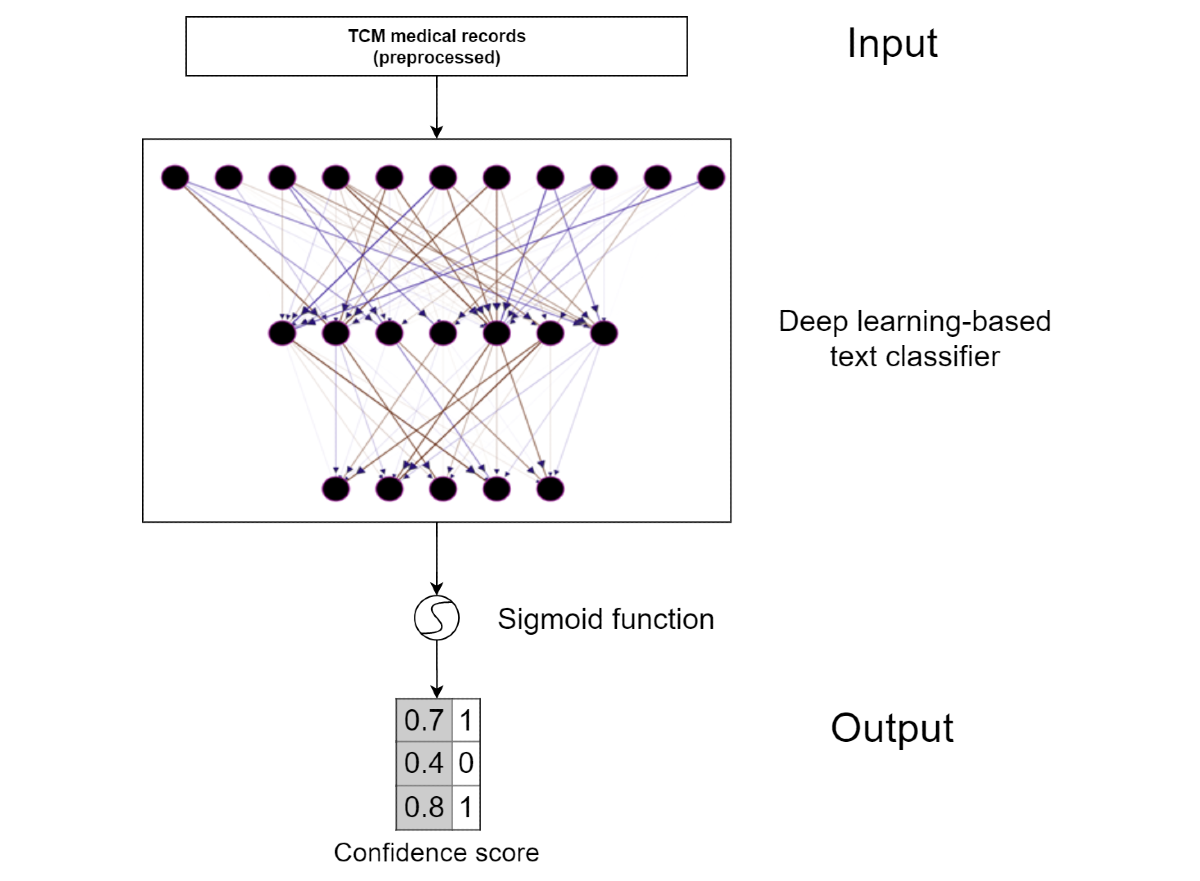
Schematic of the deep learning–based multilabel classifier.

As shown in Figure 2, the deep learning–based syndrome differentiation models consisted of a classification layer and a sigmoid activation function. The models were fed by preprocessed TCM medical records and produced a sequence of label scores corresponding to each category. If the confidence score was higher than the threshold (ie, 0.5), the category label was added to the final syndrome differentiation.

Let χ = (*x*_1_, *x*_2_, *x*_3_, …, *x*_N_) denote the *N* dimension sample space of a medical record text and Υ = (*y*_1_, *y*_2_, *y*_3_, …, *y*_m_) denote the set of lung cancer syndrome factor labels. Formally, the syndrome differentiation multilabel learning task can then be defined as follows:

The multilabel task is to learn a function *f:* χ 2^Υ^ from a given dataset ((*x*_1_, *Y*_1_), (*x*_2_, *Y*_2_), (*x*_3_, *Y*_3_), …, (*x*_N_, *Y*_N_)), where *x*_i_∈χ and *Y_i_* ⊆ Υ are the *m*-dimension label sets.

The universal approximation theorem indicates that a feed-forward deep network with a single hidden layer containing a finite number of neurons can approximate continuous functions on compact subsets under mild assumptions on the activation function [33]. In our experiment, the multilabel models with deep learning approximated the function *f:* χ 2^Υ^ and obtained the syndrome factor prediction labels in lung cancer diagnosis. Our experiment used fastText, text-convolutional neural network (Text-CNN), text-recurrent neural network (Text-RNN), recurrent convolutional neural network (RCNN), and text-hierarchical attention network (Text-HAN) models to approximate the function *f*.

For a deep learning–based multilabel classifier, the network parameters in the label matching module must be learned from a training dataset. The classifier is represented as *C*. For *N*-class multilabel classification, we used binary cross-entropy loss function and added L2 regularization to all model parameters. The total function is as follows:


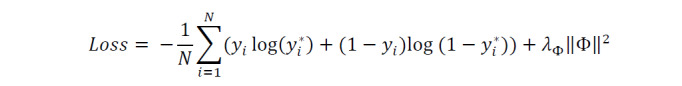
 (**1**)

where y*_i_ indicates the ground truth predictions of the *i*th sample from the training dataset, *y_i_,* is the label of the task, Φ denotes all the parameters of the model, and λ_Φ_ is the regularization hyperparameter.

We converted the multilabel classification to multiple binary classifications. The confidence score for each label in the prediction results was then obtained with multiple logistic regression models. We employed the sigmoid activation function for each label to compute the confidence score through a linear combination of each vector as

*score = sigmoid*(*w_i_O*)

(**2**)

where *O* is the output of the last layer and *w_i_* indicates the weight. In our experiment, if the confidence score for each category was >0.5, the corresponding label was included in the prediction results. If the score was <0.5, the corresponding label was not included in the prediction results.

### Deep Learning–Based Classifiers

fastText [34] was used as the baseline model in our experiments. fastText is often on par with deep neural networks in terms of classification accuracy.

The first classifier was a Text-CNN model [35]. The input word was embedded to obtain a 3D sensor. Next, a convolution layer with multiple filter widths of varying sizes and pooling layers was adopted to extract local features. We then concatenated the sigmoid function with the final fully connected layer. In this way, the Text-CNN could capture partial textual features.

The text-RNN model uses bidirectional long short term memory to extract context information and global information about sentences [36]. A traditional text-RNN uses the last hidden layer as the classification. To extract context information for each word, we used k-Max pooling for all hidden elements. We then used a fully connected layer with a sigmoid function to classify the lung cancer syndromes. In this experiment, we applied a text-RNN model with N features as inputs per sentence.

In the RCNN model [37], a recurrent structure is utilized to capture as much contextual information as possible when learning word representations. This may introduce less noise than traditional window-based neural networks. We employed a convolution layer and max pooling layer to automatically judge which words were crucial in the text classification and to capture the key components in the text. Then, the lung cancer syndrome was classified using a fully connected layer with a sigmoid function.

The Hierarchical Attention Network (HAN) [38] mirrors the document’s structure. It progressively constructs a document representation by aggregating important words into sentence representation and then aggregating important sentence representation into document representation. Therefore, two bi-directional Gate Recurrent Unit (bi-GRU) models are set to acquire the varying levels of sequence encoding. Furthermore, considering the fact that the importance of words and sentences is context-dependent, two levels of attention layers are added separately after the sequence encoder. In this way, the model can vary the amount of attention to individual words and sentences when constructing the document’s representation.

### Data Augmentation

To address possible overfitting, we added two data augmentation approaches (ie, we shuffled the sentence randomly and dropped words with a given probability). Consider the sentence ”胸片结果发现胸腔积液,去胸科医院排除结核” (*chest radiography examination shows pleural effusion, went to Chest Hospital to exclude the possibility of TB*). Using the shuffle method, the sentence may become “排除结核去胸科医院，结果发现胸腔积液胸片” (*to exclude the possibility of a TB patient going to the Chest Hospital, the examination shows pleural effusion chest radiography*); in the dropping method, it may become “胸片胸腔积液，胸科排除结核” (*chest radiography pleural effusion, Chest to exclude the possibility of TB*). During the model training batch, we used the shuffle mechanism and dropping mechanism to avoid overfitting and to ensure that the models demonstrated differences.

### Evaluation Metrics

We used evaluation metrics to measure the performance of the learning methods in our experiment. We employed micro-averaging methods to average the classes. In this way, each class could be summed and their averages could be computed.

#### Precision

Precision and recall are useful prediction success evaluation metrics when a class is imbalanced. Precision is the measure of the relevancy of the results and was computed as follows:


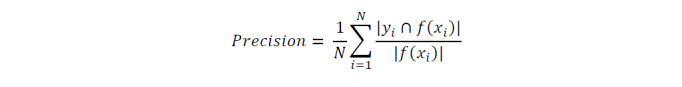
 (**3**)

where *f*(*x_i_*) is the output classifier function and *y_i_* indicates the prediction results.

#### Recall

The recall is a measure of how many relevant results are returned:


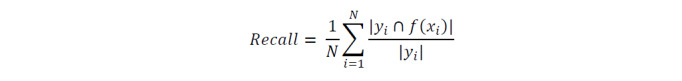
(**4**)

where *f*(*x_i_*) is the output classifier function and *y_i_* indicates the prediction results.

#### F1 Score

The F1 score is defined as the harmonic mean of the precision and recall:


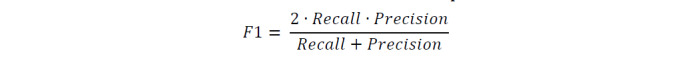
 (**5**)

#### Hamming Loss

In simplest terms, the Hamming loss is the percentage of labels that are incorrectly predicted (ie, the percentage of wrong labels). The smaller the Hamming loss value, the better the performance:


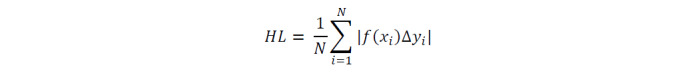
(**6**)

where *f*(*x_i_*) is the output classifier function, ∆represents the symmetry difference between the predicted label set and the true label set, and *N* indicates the class number.

#### Mean Average Precision

The mean average precision is a score that is assigned to multilabel tasks. Its value is between 0 and 1. The higher the value, the better the performance.


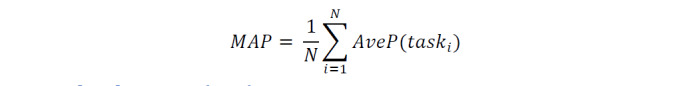
(**7**)

#### Area Under the Curve

The area under the curve (AUC) is one of the most important evaluation metrics for any classification model. The AUC refers to the area under the receiver operating characteristic curve.

## Results

### Dataset

The dataset used in the experiment consisted of 1206 clinical records of patients diagnosed with non–small cell lung cancer. The records were collected by Professor Zhongying Zhou, a renowned TCM master with expertise in lung cancer treatment. The medical records were composed of chief complaints, anamnesis, history of present illness, lab test results, four TCM examinations, and syndrome differentiation results; each visit resulted in several TCM syndrome diagnoses. Due to redundancy, the collected syndrome set required standardization, while syndromes in the dataset had distinctive personal characteristics. This causes a mapping problem in the published TCM syndrome standards that have been prevalent for decades [39]. To preserve as much of the original diagnosis as possible, we transformed each syndrome into a set of syndrome factors. These were regarded as the assembly parts of the TCM syndromes. The feasibility of this transformation has been discussed by Luo et al [40]. The splitting followed TCM syndrome factor differentiation [30]. Before factorizing, there were nearly 600 distinctive TCM syndrome labels, with 2-4 labels for each record. When the syndromes were replaced by TCM syndrome factors, only syndrome labels were left, with 2-6 labels for each record. The 12 obtained syndrome factor labels and their frequencies are shown in Table 1.

**Table 1:** TCM syndrome factors for lung cancer and their frequencies.

Syndrome factor	Frequency
Yin deficiency	1069
Qi deficiency	1052
Phlegm	1036
Stasis	1035
Cancer toxin	766
Irascibility	522
Wind	294
Thirst	79
Dampness	72
Yang deficiency	27
Qi stagnation	19
Blood deficiency	6

### Model Training

Our experimental results were obtained by 10-fold cross-validation. The entire dataset of 1206 medical records was randomly split into 10 subsets of equal size, each consisting of 120 medical records. In each of the 10 folds, a model was trained on 8 subsets, tested on 1 subset, and validated on the remaining subset. Then, the performance was averaged over the 10 folds.

For algorithm robustness and efficiency, we applied dropout to each pooling, highway, and long short term memory (LSTM) layer. For the base model, the dropout probability was 0.5, and the learning rate was set at 0.01-0.03. The hidden state dimensions in Bi-LSTM were 256. All fully connected layers contained 512 units. Moreover, the initialization network weights were sampled in a Gaussian distribution, and the bias was initialized to 0. The minimum batch size was set to 1024. To prevent overfitting during the training process, the L2 (0.00002) regularization was added for all model parameters, and we directly minimized the loss function using Adam stochastic optimization [41].

The above experiments were implemented using a computer equipped with 2 GeForce GTX 1080 Ti graphics processing units (Nvidia Corporation).

### Experimental Process

The performance of the models without and with data augmentation is shown in Tables 2 and 3. When character encoding–based representation was used as the input, the Text-HAN, RCNN, and fastText models performed best for all indicators when data augmentation was applied. Moreover, the micro-F1 scores of all five models improved. For example, in the word-encoding RCNN results with the convergence model, the F1 of RCNN with augmentation was 0.8650%-2.41% higher than that of RCNN without augmentation. The Hamming loss of RCNN with augmentation was 0.0987%-1.8% lower than that of RCNN without augmentation. These results reveal that data augmentation methods can mitigate overfitting problems.

Comparing the models, the micro-F1 scores of the Text-HAN model reached 0.8676 and 0.8751 for the character encoding–based and word encoding–based classifications, respectively; these scores are higher than those of the other four models. This may be due to the attention mechanisms and hierarchical structure, which can overcome the diffusion problem of backpropagation gradients and can detect additional information by computing the word-level and sentence-level attention. Theoretically, Text-HAN adopts two levels of attention mechanisms and hierarchical structures; thus, it can consider additional text information and ignore less relevant content when constructing the document representation.

Observing the two representation methods, the evaluation metrics denote that the models with word-encoding representation as input performed better for all indicators except for the mean average precision without data augmentation; the mean average precision of the word encoding–based RCNN with data augmentation was 10% higher than that of the character encoding–based RCNN.

To improve the classifier performance, we applied the hybrid predicting layer by linear weight after the sigmoid layer and adopted grid search methods to obtain the best hyperparameters. The hybrid results are shown in Table 4. Compared with Table 3, the model fusion approach improved the performance, especially the F1 score of the fusion model of Text-CNN, Text-RNN, and Text-HAN. The F1 score was 0.8884, which represents the best performance among the models in the experiment. Theoretically speaking, the ensemble selection used forward stepwise selection by building optimized Text-CNN, Text-RNN, and Text-HAN ensemble models. This is because the selection of features from the ensemble learning approach can exploit the advantages of all of the models to create an optimized fusion model with superior performance.

**Table 2:** Character encoding–based multilabel classification results.

Model		Precision	Recall	F1 score	Hamming loss	Mean average precision	AUC^a^
**Unaugmented**							
	fastText	0.8188	0.7923	0.8053	0.1202	0.8164	0.9211
	Text-CNN^b^	0.8327	0.8342	0.8334	0.1042	0.8634	0.9472
	Text-RNN^c^	0.8403	0.8240	0.8321	0.1231	0.8731	0.9021
	RCNN^d^	0.8467	0.8352	0.8409	0.1005	0.8842	0.9324
	Text-HAN^e^	0.8314	0.8552	0.8431	0.0990	0.8361	0.9261
**Augmented**							
	fastText	0.8447	0.8447	0.8447	0.0990	0.8752	0.9520
	Text-CNN	0.8496	0.8505	0.8500	0.1094	0.8845	0.9399
	Text-RNN	0.8267	0.8650	0.8454	0.1232	0.8010	0.9321
	RCNN	0.8652	0.8648	0.8650	0.0987	0.9056	0.9466
	Text-HAN	0.8580	0.8774	0.8676	0.0836	0.9022	0.9602

^a^AUC: area under the curve.

^b^Text-CNN: text-convolutional neural network.

^c^Text-RNN: text-recurrent neural network.

^d^RCNN: recurrent convolutional neural network.

^e^Text-HAN: text-hierarchical attention network.

**Table 3:** Word encoding–based multilabel classification results.

Model		Precision		Recall		F1 score		Hamming loss		Mean average precision		AUC^a^
**Unaugmented**												
	fastText	0.8376	0.8815		0.8590		0.040		0.8651		0.9810	
	Text-CNN^b^	0.8241	0.8520		0.8378		0.0990		0.8468		0.9395	
	Text-RNN^c^	0.8403	0.8240		0.8321		0.0960		0.8679		0.9403	
	RCNN^d^	0.8461	0.8659		0.8559		0.0832		0.8532		0.9321	
	Text-HAN^e^	0.8367	0.8505		0.8435		0.0970		0.8366		0.9260	
**Augmented**												
	fastText	0.8690	0.8760		0.8725		0.033		0.8752		0.9520	
	Text-CNN	0.8635	0.8338		0.8484		0.0886		0.8740		0.9479	
	Text-RNN	0.8377	0.8783		0.8575		0.0782		0.9052		0.9640	
	RCNN	0.8875	0.8548		0.8708		0.0532		0.9220		0.9632	
	Text-HAN	0.8648	0.8857		0.8751		0.0789		0.9210		0.9575	

^a^AUC: area under the curve.

^b^Text-CNN: text-convolutional neural network.

^c^Text-RNN: text-recurrent neural network.

^d^RCNN: recurrent convolutional neural network.

^e^Text-HAN: text-hierarchical attention network.

**Table 4:** Fusion models for multilabel classification.

Fusion model	Precision	Recall	F1 score	Hamming loss	Mean average precision	AUC^a^
Text-CNN^b^ and Text-RNN^c^	0.8898	0.8648	0.8771	0.0432	0.8836	0.9432
Text-CNN and Text-HAN^d^	0.8905	0.8732	0.8818	0.0521	0.8876	0.9524
Text-RNN and Text-HAN	0.8890	0.8635	0.8761	0.0305	0.8968	0.9687
Text-CNN, Text-RNN, and Text-HAN	0.8920	0.8890	0.8884	0.0312	0.9012	0.9618

^a^AUC: area under the curve.

^b^Text-CNN: text-convolutional neural network.

^c^Text-RNN: text-recurrent neural network.

^d^Text-HAN: text-hierarchical attention network.

## Discussion

### Principal Findings

Syndrome differentiation is the basis of rules, prescriptions, and medication in Chinese medicine. The results of syndrome differentiation directly influence clinical outcomes. Over the long history of medical practice in China, many syndrome differentiation methods have been proposed, such as six meridian, wei, qi, ying, and blood, three-energizer, viscera, and eight principles. These methods are interdependent and guide TCM clinical practice. However, the similarities and differences of these syndromes are difficult to distinguish, as disease conditions change constantly in clinical practice. The greater the number of methods for syndrome differentiation, the more chaotic the syndrome differentiation theory. This results in confusion regarding clinical syndrome differentiation. The establishment of a model to imitate syndrome differentiation has become an active research topic in TCM informatics. In recent years, statistics-based methods such as naïve Bayes, decision tree, and ensemble learning have been used in this field. However, these methods need to extract features from raw data in advance; this is a difficult task that directly influences the outcomes. Thus, reducing this influence and building a more reasonable model for TCM practice have emerged as new challenges in scientific research of clinical TCM.

The symptoms of advanced lung cancer patients are complex; therefore, their TCM diagnoses usually combine multiple syndromes. This combination is difficult to master. In this study, we ensembled end-to-end classification models based on deep learning to solve syndrome differentiation problems in TCM. This process did not require preexisting structured TCM medical records. In this study, we used syndrome factor sets instead of syndromes for the TCM diagnosis. This produces superior standardization of the various TCM lung cancer syndromes. On this basis, we established multilabel classifiers to accomplish lung cancer syndrome differentiation based on medical records collected by TCM expert Zhongying Zhou. During preprocessing, the entity-level strategy was explored due to its ability to capture partial textual features from context information. These features are implicitly learned during training. Finally, we integrated five deep learning models and conducted experiments to test their validity and benefit for TCM syndrome differentiation. Two data augmentation methods and model fusion strategies were utilized to address the overfitting problem.

### Limitations and Future Work

There are some limitations to our research. This experiment focused on a small lung cancer dataset. Although some data reinforcement methods were used, the generated data are not authentic TCM clinical data. Thus, the ensuing effects require further validation. In the future, we plan to incorporate an attention capsule network, XLNet pretrained models, and a graph neural network for lung cancer syndrome differentiation. We also plan to popularize additional TCM syndrome differentiation datasets and applications.

### Conclusion

The end-to-end models we ensembled based on deep learning can imitate syndrome differentiation from the perspective of natural language processing and may have more substantial applicability than traditional statistics-based algorithms. Therefore, these models can be embedded in TCM clinical information systems and provide clinical decision support for TCM physicians during their clinical practice, especially primary care physicians and physicians in rural areas. With the aid of our ensembled end-to-end models, TCM experiences can be learned and transferred to TCM clinical support systems, which will address the imbalance of TCM medical needs and medical supplies and provide tremendous social and economic benefit. Moreover, these end-to-end models may enable TCM institutions to efficiently transform their health record metadata into data assets.

## Abbreviations

AUC: area under the curve
BERT: Bidirectional Encoder Representations from Transformers
CNN: convolutional neural network
CRF: conditional random fields
LSTM: long short term memory
RCNN: recurrent convolutional neural network
TCM: traditional Chinese medicine
Text-CNN: text-convolutional neural network
Text-HAN: text-hierarchical attention network
Text-RNN: text-recurrent neural network
